# A Database for Learning Numbers by Visual Finger Recognition in Developmental Neuro-Robotics

**DOI:** 10.3389/fnbot.2021.619504

**Published:** 2021-03-02

**Authors:** Sergio Davies, Alexandr Lucas, Carlos Ricolfe-Viala, Alessandro Di Nuovo

**Affiliations:** ^1^Department of Computing, Sheffield Hallam University, Sheffield, United Kingdom; ^2^Department of Computer Science, The University of Sheffield, Sheffield, United Kingdom; ^3^Instituto de Automàtica e Informàtica Industrial, Universitat Politecnica de Valencia, Valencia, Spain

**Keywords:** cognitive robotics, region-based CNN, SSD, single shot detector, finger counting, iCub robot, developmental robotics, developmental neuro-robotics

## Abstract

Numerical cognition is a fundamental component of human intelligence that has not been fully understood yet. Indeed, it is a subject of research in many disciplines, e.g., neuroscience, education, cognitive and developmental psychology, philosophy of mathematics, linguistics. In Artificial Intelligence, aspects of numerical cognition have been modelled through neural networks to replicate and analytically study children behaviours. However, artificial models need to incorporate realistic sensory-motor information from the body to fully mimic the children's learning behaviours, e.g., the use of fingers to learn and manipulate numbers. To this end, this article presents a database of images, focused on number representation with fingers using both human and robot hands, which can constitute the base for building new realistic models of numerical cognition in humanoid robots, enabling a grounded learning approach in developmental autonomous agents. The article provides a benchmark analysis of the datasets in the database that are used to train, validate, and test five state-of-the art deep neural networks, which are compared for classification accuracy together with an analysis of the computational requirements of each network. The discussion highlights the trade-off between speed and precision in the detection, which is required for realistic applications in robotics.

## 1. Introduction

A novel interdisciplinary research paradigm, known as Developmental Neuro-Robotics (DNR), has been recently introduced (Cangelosi and Schlesinger, 2015; Krichmar, 2018; Di Nuovo, 2020) with the aim to create biologically plausible robots, whose control units directly model some aspect of the brain. DNR is still making its first steps, but it has been already successfully applied in the modelling of embodied word learning as well as the development of perceptual, social, language, and abstract cognition (Asada et al., 2009; Di Nuovo et al., 2013; Cangelosi et al., 2016; Cangelosi and Stramandinoli, 2018; Nocentini et al., 2019). A research area of interest for DNR is the development of numerical cognition (Di Nuovo and Jay, 2019; Di Nuovo and McClelland, 2019), which focuses on the use of fingers and gestures to support the initial learning of digits (Di Nuovo, 2020; Pecyna et al., 2020) as it has been found by numerous developmental psychology and neuro-imaging studies (Goldin-Meadow et al., 2014; Soylu et al., 2018).

The aim of the work presented in this article is to support further and more realistic studies in embodied numerical cognition. We present a novel database containing high-resolution images of both human and robot hands reproducing numbers in random positions and backgrounds. While several databases for human hand gesture recognition have been published in literature, they usually provide fixed positions and blank backgrounds, which makes it difficult to generalise in open contexts like DNR. Moreover, none of these have a focus on the development of cognitive abilities of robotics therefore they do not include robot hands. Indeed, while humanoid robot hands and fingers are designed to look and offer similar functionalities to the human's, they have structural differences and limitations because of the materials, costs, and design choices (Davis et al., 2008). These differences may impair their recognition by models trained on human hands only, therefore, it is useful to include the robot hands in the training and testing to validate the use of models in the DNR context. In addition, we provide the methodology and the experimental results to train and test on a number of neural networks, which are computationally intensive tasks. The results of this training (including accuracy in the detection of the number of fingers identifiable in the images) are presented and provide a baseline for future research with the database. Therefore, the contribution of this work is two-fold: (i) the database will facilitate researchers in developmental psychology and neuroscience to build embodied models of numerical cognition that use human or robot gestures as an input; (ii) the experiments with the deep neural network architectures will constitute a comparative benchmark, considerably reducing the amount of resources needed by machine learning researchers that want to propose new biologically inspired algorithms to support embodied learning simulations.

In the following section 1.1 we present the interdisciplinary background of the research and the motivation of the present work, then in section 1.2 we give an overview of the related work in computer vision, deep learning, and robotics. The article continues with section 2, which introduces the hands image database and the methods used to generate the images and apply silhouette extraction (section 2.1) along with an introduction to the networks presented in this experiment (section 2.2) and an overview of the process to train the networks on the finger-counting dataset (section 2.3). Then, section 3 presents the results of the training of the neural networks, with the accuracy of classification, the losses and confusion matrices and discusses the results. Finally, section 4 concludes the article.

### 1.1. Background and Motivation

Abstract concepts like mathematics are represented in the human brain with the involvement of sensory and motor cortical areas (Lakoff and Núñez, 2000). Abstract and concrete concepts are considered a continuum from the most concrete (e.g., “stone” or “water”) to the most abstract concept (e.g., “justice” or “freedom”). The learning of abstract concepts is achieved by linking them to concrete embodied perceptions, e.g., gestures, in a process of progressive abstraction (Gentner and Asmuth, 2019).

Gestures represent a form of simulated action that arise from an embodied cognitive system (Hostetter and Alibali, 2008; Tsiami et al., 2018; Chang et al., 2019). In particular, the use of hands and gestures is a very attractive method for providing natural human-computer interaction (Erol et al., 2007). Control interfaces based on gesture have been developed based on both static hand postures and dynamic motion patterns (Raheja et al., 2015; Chaudhary, 2017). Indeed, hands represent a control device with a high degree of freedom, very useful to manipulate complex machinery (Raheja et al., 2010; Wu et al., 2010) or to train systems such as surgical simulations (Badash et al., 2016).

An interesting type of gestures for computer vision are finger representations of letters and digits, which are also used as an effective form of communication in sign languages. To this extent, computers have been trained to recognise gestures from the American Sign Language (ASL), focusing on static finger spelling, used to convey names, addresses, and so on. One approach is to use depth images of the hand configuration, with further classification of the finger spelling done using evolutionary algorithms (Pugeault and Bowden, 2011), Convolutional Neural Networks (CNNs) based on AlexNet (Kang et al., 2015), or and Principal Component Analysis Network with Support Vector Machine (Aly et al., 2019). A second approach is presented by Garcia and Viesca (2016) which uses a CNN based on the GoogleNet architecture, and trained on ASL colour image datasets using transfer learning to detect static finger spelling. However, these articles do not include the gestures for number digits representation.

Number digits are at the basis of mathematics, in that they are used to count, measure, and label the fundamental workings of the universe, and form the basis of our society, from economic systems to engineering and natural sciences (Beller and Bender, 2011). The link between the body and numbers has been extensively studied in child psychology and showed that mathematics is one of the skills that can be learned through embodied cognition (Fischer et al., 2012), rather than relying only on the set of in-born child skills (Lakoff and Núñez, 2000). Numbers are taught to children from very early years using fingers to provide a spatial-numerical association (Fischer and Brugger, 2011), as well as various forms of movements, manipulations, and gestures to acquire cognitive skills (Crollen, 2011).

The tight relation between number cognition and the body is emphasised by the embodied cognition theory, which holds that many cognitive skills are acquired through embodied experiences, like movements, gestures, and manipulations, which help children in the learning of various cognitive skills by using limbs and senses to interact with the surrounding environment and other human beings (Pfeifer et al., 2007; Glenberg, 2010; Fischer and Coello, 2016; Dackermann et al., 2017). Indeed, early numerical practice is usually accompanied by gestures that are considered as a window into children's number knowledge, because children spontaneously use gestures to convey information that is not necessarily found in their speech (Goldin-Meadow, 1999).

Within the human body, a special role is attributed to hands and fingers, including a significant influence on the development of our system of counting. It is believed that we use the base 10 numbering system because we possess 10 fingers in our hands (Dantzig and Mazur, 2007). In particular, recent research on embodiment of mathematics has evidenced fingers as natural tools that play a fundamental role; from developing number sense to becoming proficient in basic arithmetic processing (Fischer et al., 2012). Also, in arithmetic tasks, fingers are used for offloading cognitive abilities by representing quantities through physical elements (Costa et al., 2011).

The simulation of numerical skills by means of computational models is a powerful tool that provides information to evaluate or compare existing theories and to make novel experimental predictions that can be tested on humans (Anderson, 2007). Computational models have the advantage of being fully specified in any implementation aspect, which makes them easily reproducible and verifiable, and they can produce detailed simulations of human performance in various situations, and, for example, experimented on with any combination of stimuli. Furthermore, models can be lesioned to simulate cognitive dysfunctions and performance can be compared to the behaviour of patients in order to gain information and insights for diagnosis and treatment that can be difficult to discover otherwise (e.g., Conti et al., 2016).

Aspects of numerical cognition have also been modeled using neural network architectures embodied in humanoid robots to mimic children learning behaviours, see Di Nuovo and Jay (2019) for an extensive review. However, for a complete emulation of human numerical cognition, artificial models need to be physically embodied, i.e., instantiated into realistic simulations of the human body that can gesture and interact with the surrounding environment, such as humanoid robots (Lungarella et al., 2003). Some development in this field has been achieved with robots being able to detect their own hands, solely using the embedded cameras (Leitner et al., 2013).

### 1.2. Related Work

All the applications covered in the introduction require a hand/finger detection system to succeed. Traditionally, these systems have been implemented using electro-mechanical or magnetic sensing devices—data gloves (Dipietro et al., 2008), which have sensors to read in real-time the hands and finger joint angles. They are usually a good source of data for human robot interaction if they do not obstruct natural hand movements. However, they are very expensive and require complicated calibration procedures.

Computer vision appears as the possible alternative solution to the hand and fingers detection problem since it is contactless, natural and done with a bare hand. However, several problems arise with this technology and it raises issues with some applications: hands may be straight or curved, partially occluded, grasping other things or other hands and they can be seen from different viewpoints.

Computer vision is computationally very expensive and in some applications special hardware has been used to enhance the identification process. Moreover, accuracy is not as good as required for some applications. In particular, if we consider self-occlusions, a complete detection of hands in images is very complex to obtain. The use of hands has limited 3D motion applications since it is very hard to extract the orientation and position of the fingers in the palm frame. To obtain hand position and orientation, 3D sensors such as Microsoft Kinect could solve this task (Raheja et al., 2013).

Some 2D computer vision algorithms for hand detection perform a silhouette analysis (Murthy and Jadon, 2009), which is a useful approach in very specific applications, but obtaining a silhouette is not trivial. Hand colour features change depending on where the hand is in the image because of the illumination and skin colour.

To perform a good image segmentation to retrieve the hand silhouette, it is necessary to control illumination and to have uniform background. In addition, skin colour influences silhouette detection since pixel intensity plays an important role in the threshold process. To resolve this colour problem, some approaches convert the image colour space from RGB (Red-Green-Blue) to HSV (Hue-Saturation-Value) or YUV (Luminance-Blue projection-Red projection), where human skin colour is easier to define.

Once the silhouette has been defined as region of interest (ROI), it is necessary to extract some scale- and time-invariant features to decide the shape or gesture of the hand. For example, convex defect detection measures ratios between convex hull area and hand silhouette area or higher distances from the silhouette to the convex hull (Xu et al., 2017).

To fix the problem of illumination, changes in skin colour and controlled backgrounds, techniques based on edge detection arise. In this case, the starting point is the gradient of the image intensity that increases the robustness against changes in lighting, colour skin and uncontrolled backgrounds. The gradient of the image highlights edges in the image.

Consequently, edge analysis allows to extract features that are heavily dependent on the hand shape and do not depend on pixel colour. The histogram of oriented gradients technique for feature extraction allows to classify gestures of bare hands with different colour skins and illumination (Chaudhary and Raheja, 2018). Orientation histogram is a technique developed by McConnell (1986) and improved by Dalal and Triggs (2005) in their work focused on human detection in images and videos.

Vision techniques have already been used in many research instances, providing some approach examples. One of these techniques allowed the identification of the hand through the use of a coloured glove, that allows segmentation of the shape of the hand within the vision field using the colour as reference (Nagi et al., 2011).

In the case of robot hands detection, the process is similarly challenging to human hand detection. Robot fingers detection is problematic because of the wide variety of shapes it can take, with problems of occlusions. An additional challenge is posed by the non-uniformity of hands and fingers material.

On the other hand, the problem of recognising hand and fingers on an image can be treated as a sub-problem in pattern recognition, a field that extensively employs artificial neural networks (ANN). In general, pattern recognition is the study of how machines interpret the surrounding environment, and how they distinguish a pattern of interest from a general background (Basu et al., 2010). Werbos (1991) estimates that approx. 80% of the work being done with ANN is related to pattern recognition tasks. A number of neural network architectures have been proposed for this finger recognition task (Abiodun et al., 2019), including Multiple Timescales Recurrent Neural Networks (MTRNN) (Antunes et al., 2018), based on Continuous Timescale Recurrent Neural Networks (CTRNN) (Funahashi and Nakamura, 1993).

Among the cognitive modellers, deep learning architectures and algorithms are becoming popular among neural networks modelers as they represent a new efficient approach to building many layers of information processing stages in deep architectures for pattern classification and for feature or representation learning (Di Nuovo et al., 2015; Sigaud and Droniou, 2016).

The deep learning approach to neural networks is inspired by the complex layered organisation of the cerebral cortex. Deep layered processing is thought to be a fundamental characteristic of cortical computation, making it a key feature in the study of human cognition. Deep learning approaches have recently been applied to the modelling of language and cognitive processing, showing how structured and abstract representations can emerge in an unsupervised way from sensory data, through generative learning in deep neural networks (for an overview see Zorzi et al., 2013). Deep learning architectures represent a new efficient approach to building many layers of information processing stages in deep architectures for pattern classification and for feature or representation learning (Salvaris et al., 2018).

Impressive results have been obtained in several areas, where deep learning architectures, such as deep belief networks (DBN) and convolutional deep neural networks, have outperformed state-of-the-art algorithms on various tasks, such as computer vision (Krizhevsky et al., 2017) and human action recognition (Ji et al., 2013).

## 2. Materials and Methods

### 2.1. Image Database Generation

Our approach to image database generation aims to replicate the approach a child would use in learning numbers and perform calculations (especially using small numbers from 1 to 5). Typically, in a learning scenario a teacher (or a parent) could show a hand representing a number to the child and say the number out loud. This would lead to the child associating the finger configuration with the meaning. In the case of a robot, one of the ways is to show the robot an image of a hand representing a number and associate the hand configuration with the number. Indeed, while a child is able to detect fingers in images innately, the robot needs to be trained for this task, which requires an image dataset specifically made for this purpose.

**Figure 1:**
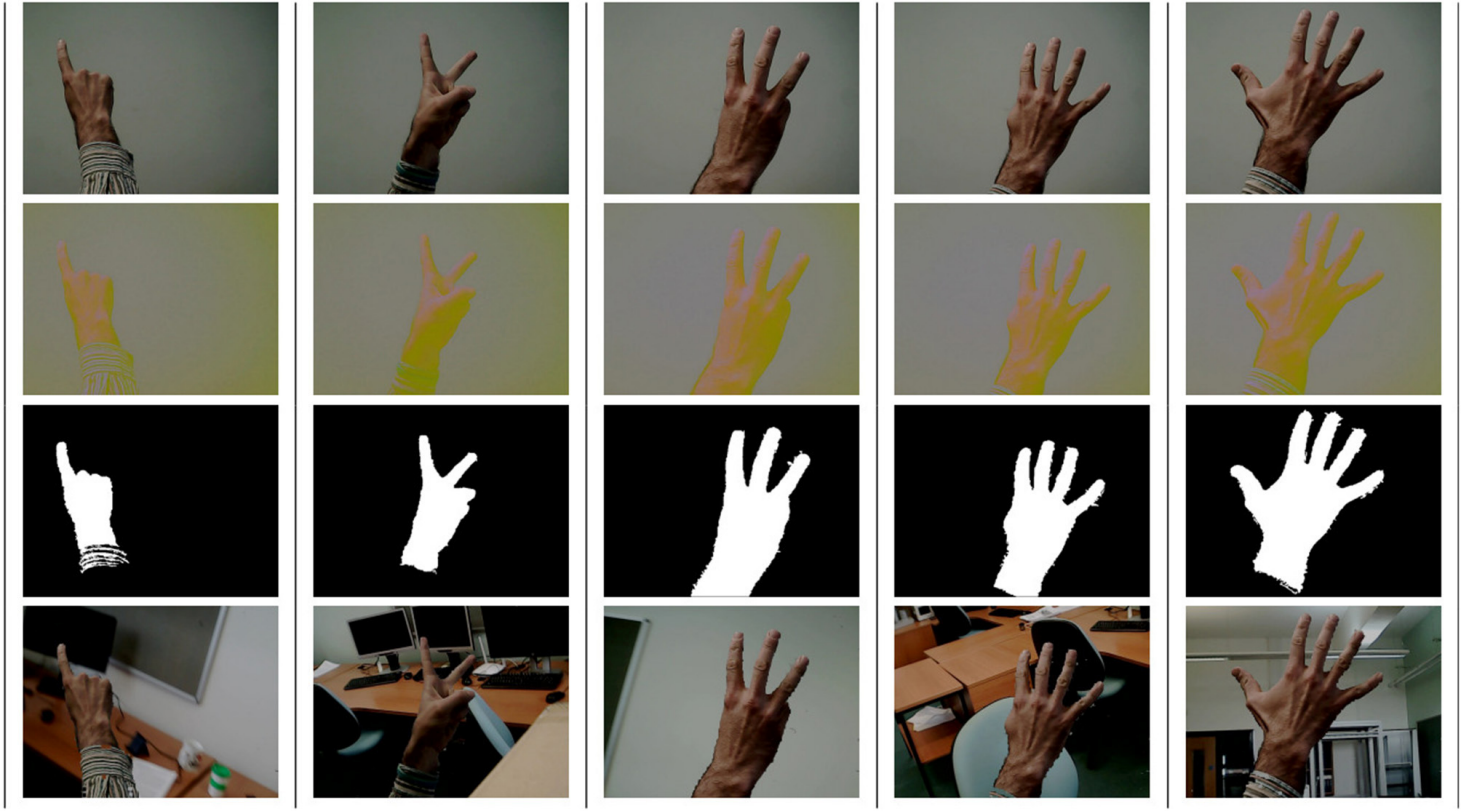
A sample of human hand images from the dataset, with columns representing numbers from 1 to 5. Each row represents one or more stages from the previous list: the first row contains original images; the second row shows the images converted from RGB to YUV; the third row shows the mask that is applied for noise removal; finally, the fourth row shows the extracted hand region using the silhouette and applied to a different background.

The database of images created for this task consists of two parts: an iCub robot's left hand in various positions with fingers showing numbers from 1 to 5, using American Sign Language, and human hands in various positions, also showing numbers from 1 to 5, generally following the ASL but also using other configurations for wider generalisation, e.g., for the 3 as shown in Figure 1. We consider the images as separate classes depending on the number shown with the hand, i.e., class “1” for hands showing the number 1, and so on. The focus on the digit representation allows to have more relevant images for this particular task, compared to other gesture or general purpose hand image datasets. In addition, all the images were made with a 640 × 480 resolution, which is typically higher than similar databases. Pictures of the iCub hand were taken using the robot's integrated cameras, while pictures of human hands were taken with a standard USB camera. The poses were randomly varied for each shot; finger representations were naturally rotated and translated within the frame of the picture simulating natural positions. The distance from the iCub's cameras to its hand was kept stable at 0.4 m, and the distance from the camera to the human hands at was approximately steady at 1.5 m, with small natural fluctuations.

Overall, we created 4,346 raw images of digit representations. The dataset with human hands has a total of 2,346 split into two sets of 1,440 and 906 pictures taken from different individuals with different poses, comprising about 300 and 200 images for each class, correspondingly. The dataset with digits represented by the iCub robot's hand has a total of 1,998 images: 1,000 taken with the left camera, with 200 images for each class with different poses, and 998 taken with the right camera. More details are in the github repository.

Image segmentation and labelling are crucial in the training process. Through these two steps, the ANN defines the region in each image where objects, in this case fingers, are present. Various applications for manual image labelling are available^1^, in which the user has to draw regions of interest (ROI) where objects in the image are. However, as the number of images to label grows, the labelling process becomes increasingly tedious and the risk of labelling errors arises. To avoid errors and simplify the annotation process, images were taken in a controlled environment with a fixed background, to allow automatic hand silhouette extraction through standard image processing techniques. A useful side effect of this process is the possibility to easily change the background as required.

The silhouette extraction and background change method for each image was done using a popular computer vision library OpenCV (Bradski, 2000). The algorithm was presented in the preliminary conference paper (Lucas et al., 2019), and comprises the following stages:

1. Pictures of hands are taken on a controlled background;
2. The RGB colour space is converted to HSV for robot hands and YUV for human hands, to highlight the hand pixels in the image;
3. Contours of all objects are detected in the HSV or YUV image;
4. The contour of the bigger object is selected as the hand silhouette;
5. Closing algorithm is applied to remove noise because of the lighting;
6. Pixel segmentation is applied to separate the original background from the hand;
7. Resulting ROI with the silhouette is saved in an “.xml” file;
8. From the hand silhouette, a new image can be created by superimposing the resulting hand pixels on the desired background image.

From all the images in the *database*, we compiled a *dataset* example, with 2, 800 images for the training, validation, testing (2, 000 − 400 − 400) of the deep learning neural networks, described in more detail in sections 2.2 and 2.3. The dataset contains images of both human and robot hands in equal proportion, sampled evenly to represent the digits 1 to 5 in various poses. Afterwards, a static data augmentation was performed by applying a random background to each image from a collection of 40 different backgrounds, but preserving the size and position of the hand on the image. The validation set was used to decide when to stop the training process; whereas the test set, never seen before by the networks, was used to evaluate the final performance of the classifiers.

**Figure 2:**
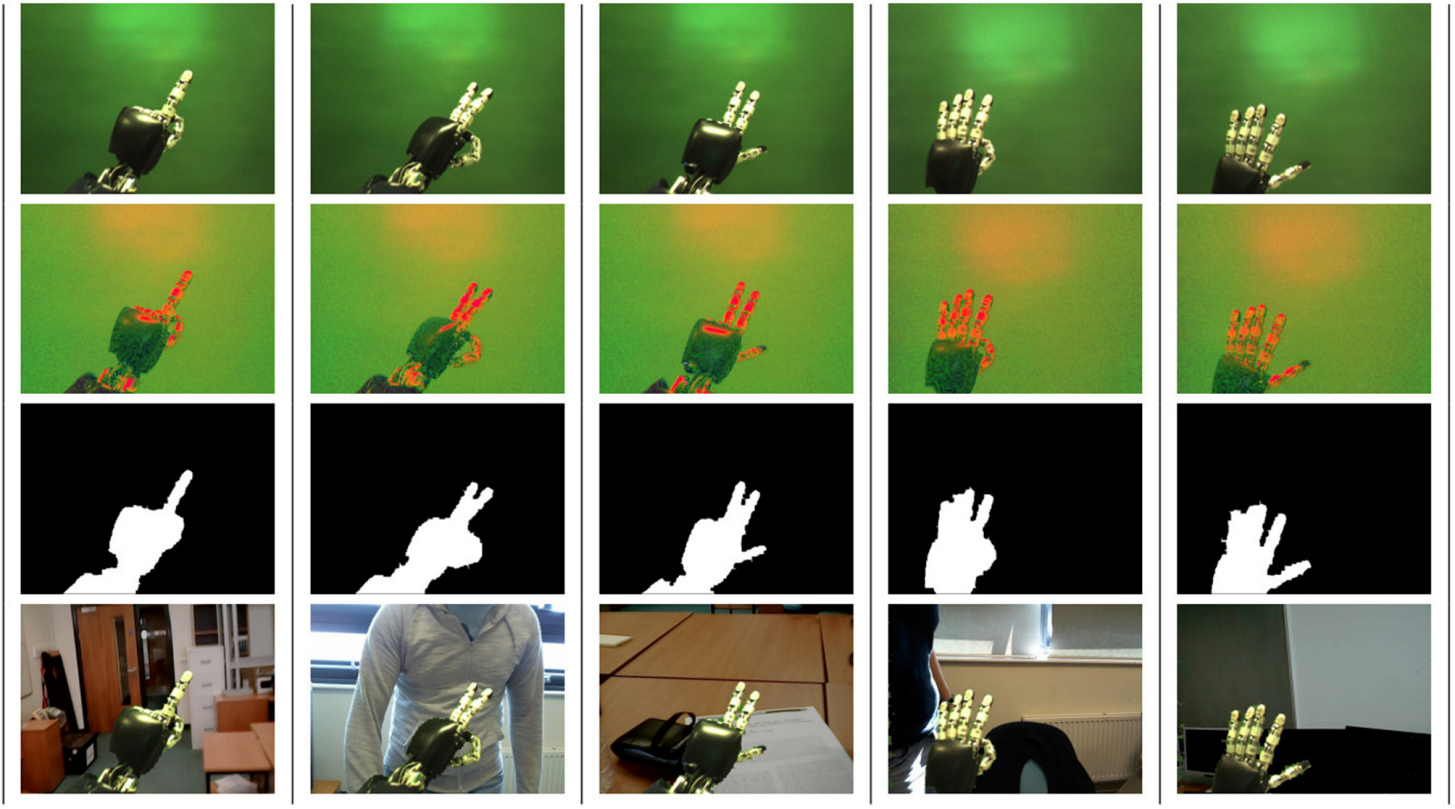
A sample of the iCub robot hand images from the dataset, with columns representing numbers from 1 to 5. Each row represents one or more stages from the previous list: the first row contains original images; the second row shows the images converted from RGB to HSV; the third row shows the mask for noise removal; finally, the fourth row shows the extracted hand region applied to a different background.

The database, along with the exact images used in the dataset and helper scripts, is publicly available on GitHub: https://github.com/EPSRC-NUMBERS/Human-Robot_Finger-Counting_Dataset. A sample of the images with the corresponding hand silhouettes is given in Figure 1, for human hand, and in Figure 2 for iCub robot hand. Note that the our implementation of the closing algorithm removes the noise only from hands and fingers, which are the focus of our recognition, but not from other parts that may be included into the silhouette contour, e.g., the arm.

### 2.2. Neural Networks and Deep Learning

In recent years, artificial neural networks (ANNs) have been successfully used in many practical applications, including computing, science, engineering, and medicine among many others (Abiodun et al., 2018). According to Haykin (2008), there are correlations in the way an artificial neural network and the human brain process information (Haykin, 1996): they both use simple units (“neurons”) interconnected working together to solve specific problems. ANNs include a set of vectors of neurons with an activation function, interconnected with weighted connections and input biases. Weights and biases can adapt and modify, following training, to fulfil a specific task. The potential of ANNs reside in their ability to be parallelised so that the computation can take place on massively parallel computers which, in turn, allows for new, more complex models to be developed. The majority of ANN applications are concerned with classification, clustering, prediction, and pattern recognition (Abiodun et al., 2019), using combinations of feed-forward and feedback neural networks architectures (Bishop, 1995).

In feed-forward networks, data, arranged in a vector, passes through a layer of neurons whose output forms the input vector of the next layer. Following this strategy and considering that an image is a 2D array, neurons in one layer arranged in a 2D array can apply a convolutional filter on an image. A convolutional filter applies a specific spatial operator that highlights features (e.g., edges) as a result. Several convolutional layers compute low-level, mid-level and high-level features of an image. A complete image classification model is a combination of convolutional and non-linearity layers, followed by several fully connected layers. With this architecture a number of models have been proposed, such as AlexNet (2012), ZF Net (2013), GoogLeNet (2014), VGGNet (2014), ResNet (2015), DenseNet (2016) (Khan et al., 2020).

Starting to build a finger detection model from scratch is an arduous task that could take a long time to reach a solution. A classification model based on neural networks has thousands of neurons with hundreds of thousands of weights and biases to tune. Adjusting all these parameters represents a very challenging task. Revising the state of the art of object detection in images, all methods are based on region proposal classification where a set of bounding boxes with a wide range of sizes and aspect ratios hypothesize object location in the image. All these bounding boxes are classified into classes with different scores. Regions are resampled to extract features with several layers of convolutional neural networks. Classification is the process where these features feed a fully connected layer and a softmax classifier (Szegedy et al., 2017, Figure 15). Processes of resampling and normalisation (Ioffe and Szegedy, 2015) between CNN layers decrease the size of the model and avoid saturations. Region-based CNNs (RCNNs) are an evolution of the AlexNet that won the LSVRC2012 image classification competition (Ren et al., 2017). Girshick et al. (2014) proposed this method to bypass going through all the regions with the objects of interest, and instead use selective search to select only a fixed number of regions. Another common architecture is Single Shot MultiBox Detector (SSD) that completely eliminates proposal generation Liu et al. (2015). MobileNets (based on SSD) are efficient convolutional neural networks for mobile and embedded vision applications (Sandler et al., 2018, Figure 4A). Inception SSD (ISSD) is an improvement of the SSD to increase its classification accuracy without affecting its speed (Chengcheng Ning et al., 2017).

To tackle the finger detection and classification problem we used a popular deep learning technique, called “transfer learning” (Razavian et al., 2014), where generic image representation descriptors extracted from a CNN model trained on one dataset, can still be highly effective when applied to a different task on a different dataset. Architectures based on CNN networks are naturally fit to implement the learning transfer approach because the convolutional layers are able to extract inherent properties from images that can be independent of the problem and, therefore, be generalised and used as a base for different classification problems (Weiss et al., 2016).

The training process needs a large dataset of labelled images where object regions in the images are defined and classes identified to adjust all the model parameters. During the last decade, many publicly available datasets have appeared, such as COCO (Lin et al., 2014), Kitti (Geiger et al., 2012, 2013), Open Images (Krasin et al., 2017), Pets (Parkhi et al., 2012). The availability of such datasets was one of the main reasons for rapid development of machine learning software libraries in the research community, with Caffe (Jia et al., 2014), TensorFlow (Abadi et al., 2016), and Keras (Chollet and others, 2015), amongst the more popular ones. These frameworks often have a collection of pre-trained models that can be used for out-of-the-box inference, or used as a base for further training algorithms, such as transfer learning.

TensorFlow, in particular, has a large collection of pre-trained object detection models on its GitHub page, the so-called “model zoo”^2^. When the project started, we chose up-to-date models from this “model zoo” that featured high accuracy relative to their size and training time, namely:

- *FRCNN1*: Faster R-CNN with Inception ResNet v2 Atrous trained on COCO dataset (version of 28th*Jan*2018)
- *FRCNN2*: Same as first, but with lower region proposal rate (version of 28th*Jan*2018)
- *FRCNN3*: Same as first, but trained on Open Image Dataset v4 (version of 12th*Dec*2018)
- *SSD*: SSD Inception v2 trained on COCO dataset (version of 28th*Jan*2018)
- *SSD Lite*: SSD Lite Mobilenet v2 trained on COCO dataset (version of 9th*May*2018).

The aim was to have a good representation in terms of accuracy/speed trade-off, with Faster R-CNN (*FRCNN*) models featuring very good classification accuracy but being rather slow; and Single-Shot Detector (*SSD*) models, on other hand (especially “light”) being generally faster but less accurate. Additionally, during a preliminary study, the Faster R-CNN with Neural Architecture Search (NAS) framework was trialled as well, but the training turned out to be prohibitively slow (1 epoch ≈ 1 week computational effort), therefore this architecture was not considered in the final list of architectures for comparison.

### 2.3. Training the Neural Networks and Evaluation Metrics

The training, validation, and test sets comprised 2,000, 400, and 400 images, respectively. Images for the training and validation sets were combined with the corresponding ROI data into two Tensorflow “.record” files to perform hand and finger detection (with boxes as output) and classification based on the digit being shown, represented by the position of the hand and fingers. For *FRCNN* networks, the training batch size was set to one image (which, incidentally, coincided with the default value), to resemble how a human child would be trained, e.g., being shown one gesture at a time. In this regime, therefore, one epoch would amount to 2,000 training steps. The batch size for *SSD* architectures was kept at the default value of 24 images, thus an epoch would take approximately 83 training steps.

Approximately after each epoch, the model checkpoint was saved and the classifier was run on the validation set to compute total loss and mean Average Precision (mAP), the two measures that were used to evaluate the classification performance. Total loss is the weighted sum of classification loss (softmax function) and localisation loss, which for *SSD* is a weighted sigmoid function. For *SSD* networks the total loss can take values larger than one. Mean Average Precision (mAP) is a metric that computes the average precision value of classification. The values for both of these metrics were generated by the default tools provided by the TensorFlow 1.12.0 framework. Table 1 presents some of the more important hyperparameters and settings in the training protocol.

**Table 1:** Network training hyperparameters.

**Network Training hyperparameters**	***FRCNN1***	***FRCNN2***	***FRCNN3***	***SSD***	***SSD Lite***
Batch size	1 image			24 images	
Learning rate	0.0003		0.0006	0.0004	
Classification layer transfer function	Softmax			Sigmoid	
Data augmentation	Random horizontal flip			Random horizontal flip and random crop	

**Figure 3:**
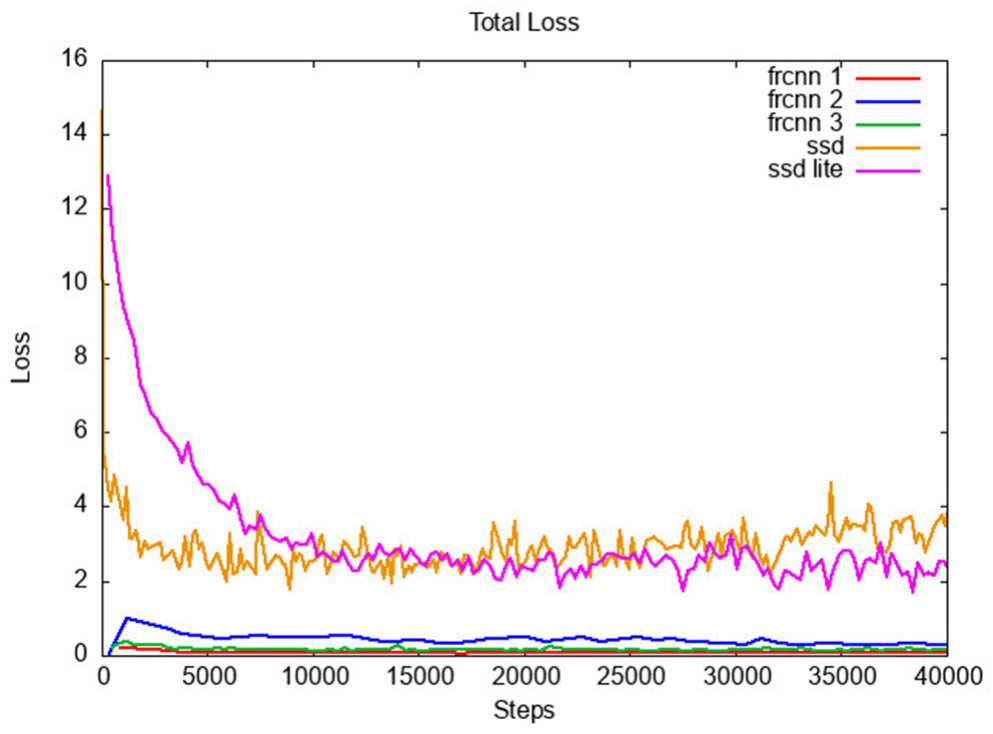
Loss function for all networks, in relation to training steps.

**Figure 4:**
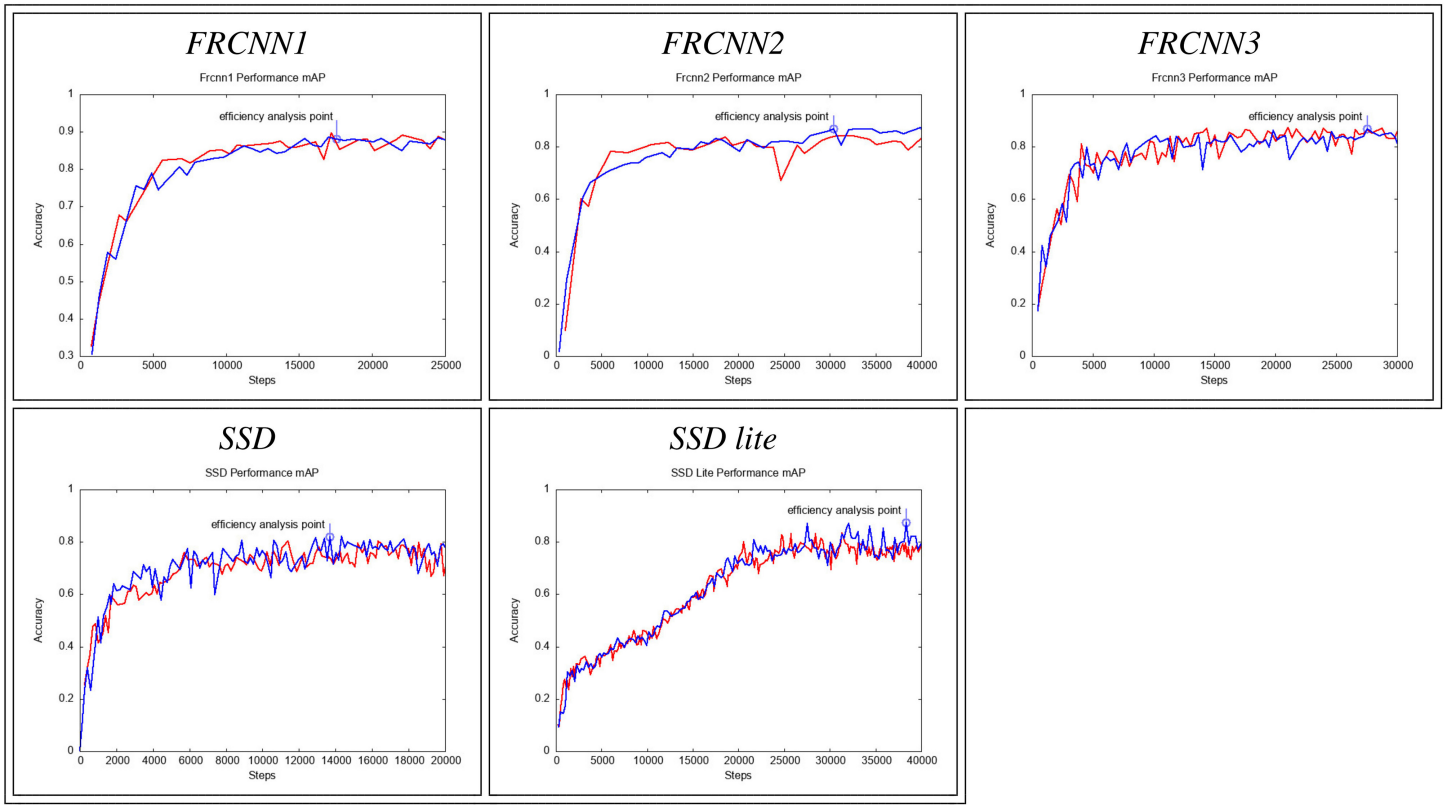
Mean Average Precision (mAP, y-axis) of the 5 networks plotted against the training steps (x-axis), as evaluated on the validation set. Blue circles represent model checkpoints with the highest mAP, which were then used for classifier evaluation on the test set.

Each of the networks was trained twice, to analyse the range of variation of the classification accuracy with the number of training steps, to understand if a trend in the learning process is present. **Figure 4** presents the progression of the likelihood/confidence of the classification task on the validation set, after an increasing number of steps the network has been trained with the training set. As training progresses, the network improves its classification abilities until a point when classification on the validation dataset does not improve further. Figure 3 shows how the model losses decrease with the number of training steps. The confusion matrices and the statistics related to the confusion elements are computed starting from the definition of the confusion matrix:

$$\begin{array}{c} C=Actual\begin{array}{c} Classified\\ \begin{array}{ccc} c_{11} & \cdots & c_{1n}\\ \vdots & \ddots & \vdots \\ c_{n1} & \cdots & c_{nn} \end{array} \end{array} \end{array}$$

Each element of this matrix identifies how many images of a given class were assigned to each of the possible network output classes. Columns identify classes available for the network outputs (one, two, three, four, five, and non-classified–N.C.). Rows identify the actual class to which an image belongs, considering the number of fingers shown in the image. The optimal confusion matrix is the one that displays 100% values only on the main diagonal, so that each image is correctly placed into the appropriate network output class, and no cross-inference took place.

The confusion elements for each class are computed using these definitions:

**Table:** 

True Positives:	*tp*_*i*_ = *c*_*ii*_
False Positives:	$fp_{i}=\overset{n}{\underset{l=1}{\sum }}\left(c_{li}-tp_{i}\right)$
False Negatives:	$fn_{i}=\overset{n}{\underset{l=1}{\sum }}\left(c_{il}-tp_{i}\right)$
True Negatives:	$tn_{i}=\overset{n}{\underset{l=1}{\sum }}\overset{n}{\underset{k=1}{\sum }}\left(c_{lk}-tp_{i}-fp_{i}-fn_{i}\right)$
Positives:	*P*_*i*_= number of positive cases for the *i*-th class
Negatives:	*N*_*i*_= number of negative cases for the *i*-th class

Confusion statistics are computed following the definitions in Di Nuovo et al. (2018):

**Table:** 

**Name**	**Formula**	**Description**
*Accuracy*	$\frac{tp_{i}+tn_{i}}{P_{i}+N_{i}}$	Proximity of the classification results to the true values. It evaluates the overall performance of classification
*Precision*	$\frac{tp_{i}}{tp_{i}+fp_{i}}$	Positive predicted value. This indicates the reliability of classification
*Negative Prediction*	$\frac{tn_{i}}{tn_{i}+fn_{i}}$	Reliability of classification of distractions
*Sensitivity*	$\frac{tp_{i}}{P_{i}}$	Focuses on how good is the performance in classifying attention
*Specificity*	$\frac{tn_{i}}{N_{i}}$	Evaluates the performance in classifying distractions

## 3. Results

For the analysis of the performance of all the networks under analysis we selected a stage of the training where a full snapshot of the network status was available and for which the loss was as close as possible to the lowest point while the accuracy was as close as possible to its maximum value, as observed on the validation test (Figure 4).

Under these constrains, networks were analysed at the following training steps:

- *FRCNN1*: Training step = 17594, Mean Average Precision (mAP) ≈ 0.88, Loss ≈ 0.065
- *FRCNN2*: Training step = 30429, Mean Average Precision (mAP) ≈ 0.87, Loss ≈ 0.28
- *FRCNN3*: Training step = 27548, Mean Average Precision (mAP) ≈ 0.87, Loss ≈ 0.134
- *SSD*: Training step = 13700, Mean Average Precision (mAP) ≈ 0.82, Loss ≈ 1.937
- *SSD Lite*: Training step = 38394, Mean Average Precision (mAP) ≈ 0.874, Loss ≈ 1.7.

Finally, an averaged computational effort per step for each network model is presented in Table 2. This table highlights the average time required for each network to classify an image (either from the validation or the test set), and to train on a single image, as measured on a workstation equipped with NVIDIA GeForce GTX 1080 Ti GPU.

**Table 2:** Average time required by each network to classify or train on an image.

**Time per image Network**	**Classification (validation set)**	**Classification (test set)**	**Training**
*FRCNN1*	≈ 0.5*s*	≈ 0.5*s*	≈ 1.12*s*
*FRCNN2*	≈ 0.23*s*	≈ 0.23*s*	≈ 0.71*s*
*FRCNN3*	≈ 0.3*s*	≈ 0.3*s*	≈ 0.88*s*
*SSD*	≈ 0.04*s*	≈ 0.04*s*	≈ 0.15*s*
*SSD Lite*	≈ 0.03*s*	≈ 0.03*s*	≈ 0.16*s*

Network performances are presented through confusion matrices (Tables 3, 4). These report how images belonging to each class are classified by each network. On each row there are the actual image classes. On the columns there are the classes predicted by each of the neural networks, in addition to an “NC” (“Non classified” column), in case the neural network is unable to classify the image.

**Table 3:** Results of the detection using the five example networks.

		***FRCNN1***						***FRCNN2***						***FRCNN3***						***SSD***						***SSD Lite***					
		**Fingers detected**						**Fingers detected**						**Fingers detected**						**Fingers detected**						**Fingers detected**					
**All values are in %**		**1**	**2**	**3**	**4**	**5**	**NC**	**1**	**2**	**3**	**4**	**5**	**NC**	**1**	**2**	**3**	**4**	**5**	**NC**	**1**	**2**	**3**	**4**	**5**	**NC**	**1**	**2**	**3**	**4**	**5**	**NC**
Actual number Signed	1	**100**	0	0	0	0	0	**97.5**	2.5	0	0	0	0	**100**	0	0	0	0	0	**90**	8.75	0	1.25	0	0	**81.25**	16.25	0	2.5	0	0
	2	2.5	**97.5**	0	0	0	0	0	**100**	0	0	0	0	1.25	**97.5**	1.25	0	0	0	1.25	**86.25**	5	6.25	1.25	0	3.75	**86.25**	6.25	3.75	0	0
	3	0	17.5	**82.5**	0	0	0	0	17.5	**75**	7.5	0	0	0	12.5	**83.75**	3.75	0	0	0	3.75	**76.25**	15	5	0	0	2.5	**80**	16.25	1.25	0
	4	0	0	3.75	**88.75**	7.5	0	0	0	1.25	**90**	8.75	0	0	0	6.25	**73.75**	20	0	0	0	1.25	**63.75**	35	0	0	0	8.75	**50**	41.25	0
	5	0	2.5	13.75	16.25	**67.5**	0	0	1.25	1.25	13.75	**83.75**	0	0	0	3.75	6.25	**90**	0	0	0	0	2.5	**97.5**	0	0	0	5	6.25	**88.75**	0
Accuracy		99.5	95.5	93	94.5	92	–	99.5	95.75	94.5	93.75	95	–	99.75	97	94.5	92.75	94	–	97.75	94.75	94	87.75	91.25	–	95.5	93.5	92	84.25	89.25	–
(Average)		94.9						95.7						95.6						93.1						90.9					
Precision		97.56	82.98	82.5	84.52	90	–	100	82.47	96.77	80.9	90.54	–	98.77	88.64	88.16	88.06	81.82	–	98.63	87.34	92.42	71.83	70.27	–	95.59	82.14	80	63.49	67.62	–
(Average)		87.512						90.136						89.09						84.098						77.768					
Negative Prediction		100	99.35	95.63	97.15	92.35	–	99.38	100	94.08	97.43	96.01	–	100	99.36	95.99	93.69	97.44	–	97.55	96.57	94.31	91.19	99.31	–	95.48	96.52	95	88.13	96.95	–
(Average)		96.896						97.38						97.296						95.786						94.416					
Sensitivity		100	97.5	82.5	88.75	67.5	–	97.5	100	75	90	83.75	–	100	97.5	83.75	73.75	90	–	90	86.25	76.25	63.75	97.5	–	81.25	86.25	80	50	88.75	–
(Average)		87.25						89.25						89						82.75						77.25					
Specificity		99.38	95	95.63	95.94	98.13	–	100	94.69	99.38	94.69	97.81	–	99.69	96.88	97.19	97.5	95	–	99.69	96.88	98.44	93.75	89.69	–	99.06	95.31	95	92.81	89.38	–
(Average)		96.816						97.314						97.252						95.69						94.312					

*The images used for these results belong to the “validation” set. The colours are for visualisation purposes: greener colors represent better performance. The “NC” columns identify how many images have not been classified by the network. Bold values represent correct interpretations of the images in the set by each of the networks: images of one fingers correctly interpreted as 1, and so on*.

**Table 4:** Results of the detection using the five example networks.

		***FRCNN1***						***FRCNN2***						***FRCNN3***						***SSD***						***SSD Lite***					
		**Fingers detected**						**Fingers detected**						**Fingers detected**						**Fingers detected**						**Fingers detected**					
**All values are in %**		**1**	**2**	**3**	**4**	**5**	**NC**	**1**	**2**	**3**	**4**	**5**	**NC**	**1**	**2**	**3**	**4**	**5**	**NC**	**1**	**2**	**3**	**4**	**5**	**NC**	**1**	**2**	**3**	**4**	**5**	**NC**
Actual number Signed	1	**100**	0	0	0	0	0	**77.5**	15	0	0	0	7.5	**86.25**	6.25	0	1.25	0	6.25	**31.25**	13.75	6.25	2.5	1.25	45	**23.75**	2.5	0	6.25	0	67.5
	2	31.25	**63.75**	5	0	0	0	7.5	**82.5**	0	8.75	0	1.25	10	**68.75**	12.5	7.5	0	1.25	0	**45**	16.25	11.25	2.5	25	1.25	**23.75**	8.75	20	0	46.25
	3	8.75	20	**58.75**	12.5	0	0	0	32.5	**36.25**	31.25	0	0	2.5	16.25	**57.5**	21.25	0	2.5	0	6.25	**57.5**	1.25	11.25	23.75	0	0	**28.75**	6.25	1.25	63.75
	4	6.25	3.75	0	**88.75**	1.25	0	2.5	2.5	1.25	**92.5**	0	1.25	2.5	1.25	1.25	**87.5**	1.25	6.25	2.5	5	1.25	**60**	13.75	17.5	0	1.25	0	**51.25**	0	47.5
	5	8.75	6.25	3.75	42.5	**38.75**	0	0	12.5	6.25	62.5	**17.5**	1.25	2.5	3.75	13.75	53.75	**18.75**	7.5	1.25	11.25	7.5	12.5	**47.5**	20	0	1.25	5	13.75	**13.75**	66.25
Accuracy		89	86.75	90	86.75	87.5	–	93.5	84	85.75	78	83.5	–	93.75	88.25	86	80.75	83.5	–	85.5	81.75	85.25	86.5	83.75	–	84.5	83.75	83	81	82.5	–
(Average)		88						84.95						86.45						84.55						82.95					
Precision		64.52	68	87.04	61.74	96.88	–	88.57	56.9	82.86	47.44	100	–	83.13	71.43	67.65	51.09	93.75	–	89.29	55.38	64.79	68.57	62.3	–	95	82.61	67.65	52.56	91.67	–
(Average)		75.636						75.154						73.41						68.066						77.898					
Negative Prediction		100	91.08	90.46	96.84	86.68	–	94.55	95.07	86.03	97.54	82.9	–	96.53	92.26	89.76	96.2	83.07	–	85.22	86.87	89.67	90.3	87.61	–	83.95	83.82	84.43	87.89	82.22	–
(Average)		93.012						91.218						91.564						87.934						84.462					
Sensitivity		100	63.75	58.75	88.75	38.75	–	77.5	82.5	36.25	92.5	17.5	–	86.25	68.75	57.5	87.5	18.75	–	31.25	45	57.5	60	47.5	–	23.75	23.75	28.75	51.25	13.75	–
(Average)		70						61.25						63.75						48.25						28.25					
Specificity		86.25	92.5	97.81	86.25	99.69	–	97.5	84.38	98.13	74.38	100	–	95.63	93.13	93.13	79.06	99.69	–	99.06	90.94	92.19	93.13	92.81	–	99.69	98.75	96.56	88.44	99.69	–
(Average)		92.5						90.878						92.128						93.626						96.626					

*The images used for these results belong to the “test” set. The colours are for visualisation purposes: greener colors represent better performance. The “NC” columns identify how many images have not been classified by the network. Bold values represent correct interpretations of the images in the set by each of the networks: images of one fingers correctly interpreted as 1, and so on*.

The confusion matrices show that, on average, there is more confusion with a higher number of fingers. This is generally due to the occlusions among fingers in the image (e.g., see **Figure 6**), and often even a human eye may be incorrectly counting fingers in such conditions. Specifically, in the images showing two, three, four, and five fingers, it is possible to notice that the overlap requires also a human viewer to observe carefully to correctly identify all fingers. Besides the case of overlapping fingers, other situations in which a network may be unable to classify images is if it is unable to detect fingers, perhaps due to position, size, or inclination of the hand. In these cases the image is reported as “Non Classified” (“NC” column).

If we consider the ability of the network to classify images, it is possible to notice that network *FRCNN1* always reports 0 in the “NC” column, for any image that has been presented (both for validation and test images). The same network has also the highest statistics of all the networks for the test set, with an averaged accuracy of ≈ 88% and a mean precision of ≈ 75.6%. However, on the image validation set, the network *FRCNN2* has the highest averaged statistics, with a mean accuracy of ≈ 95.7% and a mean precision of ≈ 90.1%.

Analysing the results of the confusion matrices on the test set (Table 4) it is possible to notice that the results of classification of 4 and especially 5 fingers is prone to misclassification. In particular the images with 5 fingers are more often classified as 4-finger images, because one of the vision of one of the finger may be occluded in some poses. This is often due to the misinterpretation of the position of the thumb: in fact, the features extracted from these two classes are not enough to differentiate reliably between the two, as they differ only by the position of a single phalanx. In *FRCNN2, FRCNN3*, and *SSD Lite*, the detection of images with five fingers has a sensitivity below what is expected from a random choice among 5 classes (20% expected).

The classification of robotics finger representations proved to be more challenging than the human ones. This is evidenced by Table 5, which shows the number of human and robotic images in the test set that are correctly classified by all the networks considered. We would clarify that each network classified a different subset of images in the test set, Table 5 considers only the subset of those that were correctly classified by all networks. Out of 400 test images, 80 per class (40 robot + 40 human), a total of 173 images are correctly classified by all networks, of which 151 (75.5%) were from the human dataset and only 22 (11%) from the robotic dataset. In fact, in the test set, there are no images of robotic fingers representing number 5 that were correctly classified by all networks under analysis, while just a few robotic images of numbers 2, 3, and 4 are correctly recognised by all networks.

**Table 5:** Examples of images from the subset of test images that are correctly classified by all networks.

	**One finger**	**Two fingers**	**Three fingers**	**Four fingers**	**Five fingers**
Human hands	36	34	15	34	32
Robot hands	14	1	2	5	0

**Figure 5:**
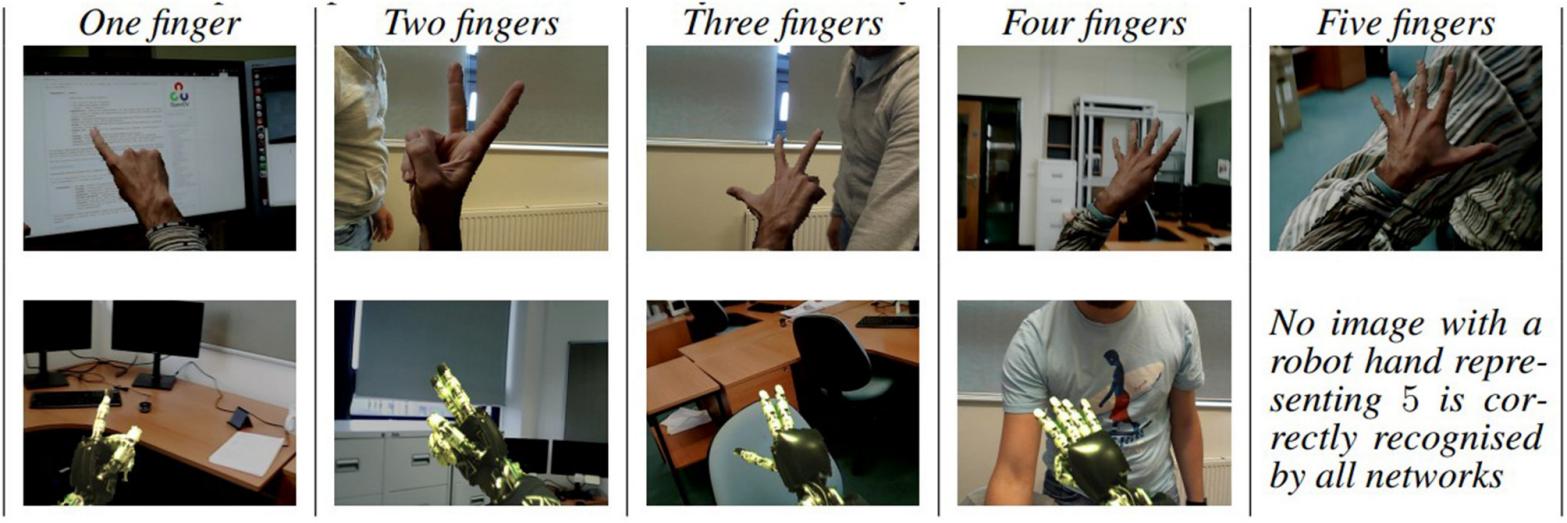
Examples of from the subset of test images that are correctly classified by all networks.

**Figure 6:**
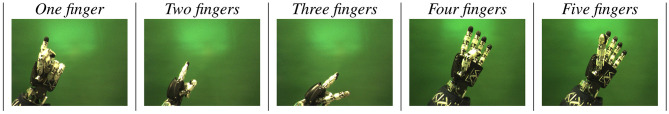
Pictures showing examples of hand positions that are difficult for networks to classify unambiguously. The “one finger” image is classified by *FRCNN3* as having four fingers on display because of the palm position. The “two fingers” and “three fingers” images provide an example of overlap, hard to identify the correct number also for the human eye. The “four fingers” and “five fingers” images show subtle variation in the hand position that may easily confuse the network.

Figure 5 presents examples of the subset of the test images that were successfully classified by all networks. These examples are for each number and from both the human and the robot datasets. Note that there was no image of 5 robot fingers that was correctly classified by all networks. Figure 6 presents a set of images, one per class, showing robot hand poses that were wrongly classified by all networks because of the perspective in which fingers cover each other and, therefore, make them difficult to count.

The network models were also tested in real time, using a webcam to capture the image of human hands. It was noted that the presence of faces and other body parts adversely affected the classification due to a large number of false positives coming from these regions of the image. It was speculated that the models were not trained sufficiently to differentiate features extracted from hands, as opposed to similarly coloured objects, such as arms or faces. To remedy this, a number of “negative” background images were added to the pool, showing people with their arms, but not their hands with fingers. The networks were trained not to classify these features. During further testing, this procedure demonstrated improvement in the classification results.

In Figure 3 and Table 2 it is possible to notice that the two classes of neural networks differentiate significantly in training, both in terms of loss and execution time per step. While *FRCNN* networks require above 200*ms* to classify an image, and above 700*ms* for a training step on a single image, they show a training loss constantly below 2. On the contrary, networks based on the single-shot detector (*SSD*) architecture are consistently below the 200*msecs* mark for both training and classification for a single image, but the loss is, in average, above 2. This is a consequence of the network size: *FRCNN* networks are larger than *SSD* networks, and therefore they adapt better to previously unseen input, but they are computationally more expensive, both for training and for classification.

The confusion matrices for both the validation and test image sets highlight that the statistics for the convolutional neural networks based on Faster Region-Proposal (*FRCNN*) perform consistently better than the networks based on the Single-Shot Detector *SSD* architecture.

In the graphs showing the progression of the classification precision (Figure 4) it is possible to notice that in four of them the trend tends very quickly to an asymptote, and then fine tunes around it with the following training iterations. The *SSD Lite* network, instead has a different trend, almost linear at the beginning. This peculiar behaviour is estimated to derive from the size of the network: the *SSD Lite* network is smaller than all the other neural networks used for comparison.

In addition, both the networks based on *SSD* and the *FRCNN3* have wider variation on accuracy, depending on the training step. This requires a careful consideration of when to stop the training to obtain a reliable classification, but at the same time the training speed is higher than other networks. The *SSD* is the network that reaches more quickly an optimal training point, while the *SSD Lite* network is the one that reaches it later.

## 4. Discussion

This article has introduced a novel image database that supports recognition of finger digits configuration to be used in a developmental neuro-robotics research environment. This open access database comprises 4,346 images of both human and robot hands made with 640 × 480 resolution (higher than typical datasets), and is tailored to digit representation using fingers, as opposed to manipulation, grasping, or finger spelling. The images are made in a controlled environment with a known background, which eases further manipulation. This new database complements what is already in the public domain and expands the tool-set available for developmental neuro-robotics and AI research.

In addition, the article provides a comparative analysis of the performance of 5 state-of-the-art deep learning artificial neural networks with this database. The benchmark is meant to act as a quick guide for follow-up research in deep learning and neuro-robotics. Future researchers can benefit and benchmark baseline by comparing their work with the results presented in this paper, enabling them to save time and resources.

The comparative analysis of the deep learning networks show a spectrum of performance, with the *FRCNN1* network being the slowest to perform the train (≳ 1 *s*/*image*) and to perform the classification, but the most accurate (accuracy ≈ 88% and precision ≈ 75.6%). At the other end of the spectrum, the *SSD Lite* network has the fastest training (≲ 0.2 *s*/*image*) and classification time, but the lowest accuracy (accuracy ≈ 82% and precision ≈ 77.9%).

To summarise, the 5 networks provide a range for trade-off between speed of training and classification and their ability to classify previously unseen images: *FRCNN* networks are slower, and perform better, whereas *SSD* networks are faster, but their ability to classify previously unseen images is reduced.

## Data Availability Statement

As described in previous sections, both the data and the code used to generate the image dataset are freely available on GitHub (URL: https://github.com/EPSRC-NUMBERS/Human-Robot_Finger-Counting_Dataset) and through the DOI http://doi.org/10.17032/shu-180017 pointing at the GitHub NUMBERS project repositories (URL: https://github.com/465EPSRC-NUMBERS).

## Author Contributions

SD set up the article structure, prepared the first draft, and some of the images. AL contributed portions of the text, results of experiments, image capturing with robotic hands, image manipulation for the database, and reviews of the article. CR-V contributed images captured for the database and images of human hands published in this paper. AD conceptualised and led the study, supervised the realisation of project, contributed to the abstract, background and discussion sections. All authors reviewed and approved the text.

## Conflict of Interest

The authors declare that the research was conducted in the absence of any commercial or financial relationships that could be construed as a potential conflict of interest.

## Footnotes

^1^List of manual image annotation tools from Wikipedia: https://en.wikipedia.org/wiki/List_of_manual_image_annotation_tools.

^2^TensorFlow 1 Detection Model Zoo: https://github.com/tensorflow/models/blob/master/research/object_detection/g3doc/tf1_detection_zoo.md.
